# Project Stakeholder Management in the Clinical Research Environment: How to Do it Right

**DOI:** 10.3389/fpsyt.2015.00071

**Published:** 2015-05-18

**Authors:** Seithikurippu R. Pandi-Perumal, Sohel Akhter, Ferdinard Zizi, Girardin Jean-Louis, Chellamuthu Ramasubramanian, R. Edward Freeman, Meera Narasimhan

**Affiliations:** ^1^Department of Population Health, New York University Medical Center, New York, NY, USA; ^2^Department of Management, Zicklin School of Business, Baruch College, New York, NY, USA; ^3^District Mental Health Programme, Madurai Medical College, Madurai, India; ^4^Division of Community Psychiatry, M. S. Chellamuthu Trust and Research Foundation, Madurai, India; ^5^University of Virginia Darden School of Business, Charlottesville, VA, USA; ^6^Department of Neuropsychiatry and Behavioral Science, University of South Carolina School of Medicine, Columbia, SC, USA; ^7^University of South Carolina, Columbia, SC, USA

**Keywords:** PCORI, PMBOK, PMI, clinical research, code of ethics, professional conduct, project stakeholder management

## Abstract

This review introduces a conceptual framework for understanding stakeholder management (ShM) in the clinical and community-based research environment. In recent years, an evolution in practice has occurred in many applicants for public and non-governmental funding of public health research in hospital settings. Community health research projects are inherently complex, have sought to involve patients and other stakeholders in the center of the research process. Substantial evidence has now been provided that stakeholder involvement is essential for management effectiveness in clinical research. Feedback from stakeholders has critical value for research managers inasmuch as it alerts them to the social, environmental, and ethical implications of research activities. Additionally, those who are directly affected by program development and clinical research, the patients, their families, and others, almost universally have a strong motivation to be involved in the planning and execution of new program changes. The current overview introduces a conceptual framework for ShM in the clinical research environment and offers practical suggestions for fostering meaningful stakeholder engagement. The fifth edition of PMBOK^®^ of the Project Management Institute, has served as basis for many of the suggested guidelines that are put forward in this article.

A true architect is not an artist but an optimistic realist. They take a diverse number of stakeholders, extract needs, concerns, and dreams, and then create a beautiful yet tangible solution that is loved by the users and the community at large. We create vessels in which life happens

– Cameron Sinclair (26)

In recent years, a revolution in thinking about organizational management and decision making has taken place. Increasingly, programs have been incorporated into organizations, typically private sector corporations or government agencies, which have sought to involve “stakeholders” in management decision making. Stakeholders are the customers, suppliers, the general public, and any other group, which are likely to be affected by the organization’s ultimate decisions. The process of incorporating the ideas and input from these groups has been termed “stakeholder engagement.” It reflects an increasingly accepted attitude that organizations not only have an ethical obligation to involve the participation of stakeholders in their collective activity but also in so doing their overall organizational effectiveness will be enhanced. While certain generalizations in the application of this philosophy are constant, minor variations also exist, which reflect the specific goals that the organization is pursuing. In this review, the application of stakeholder engagement in clinical research settings, particularly in hospitals or university research centers, is considered.

According to the Institute of Medicine (IOM), the purpose of comparative effectiveness research (CER) is, “to assist consumers, clinicians, purchasers, and policy makers to make informed decisions that will improve healthcare at both the individual and population level” (1). The Kellogg Commission report defines *engagement* as follows: “By ‘*engagement*’ we refer to institutions that have redesigned their teaching, research, and extension and service functions to become even more sympathetically and productively involved with their communities, however, community may be defined” (2). Hospitals and research centers are increasingly taking deliberate steps to include their broader constituencies in project management decision making and to seek their input at an early stage of the research or program implementation process. The term “*community engagement*,” can be defined as, “the process of working collaboratively with and through groups of people affiliated by geographic proximity, special interest, or similar situations to address issues affecting the well-being of those people” [(3), p.3]. It has been noted that traditional models of research which view study subjects or targets of program development as passive audiences may result in research findings that are poorly aligned with the information needs of real-world decision makers (4, 5). An additional impetus for this interest has been the Patient Protection and Affordable Care Act of 2010, which was enacted to promote patient engagement. The purpose of the act has been to help patients, clinicians, purchasers, and policy makers make better informed health decisions by “advancing the quality and relevance of evidence about how to prevent, diagnose, treat, monitor, and manage diseases, disorders, and other health conditions.”

The key focus in the process of stakeholder engagement is of course the stakeholder. Freeman (6): 46 defined *stakeholder* as, “any group or individual who can affect or is affected by the achievement of the organization’s objectives.” According to the project management institute (PMI), the term stakeholder refers to, “an individual, group, or organization, who may affect, be affected by, or perceive itself to be affected by a decision, activity, or outcome of a project” (7). In other words, almost any individual or group of individuals with an interest or stake in a consensus-building process thereby the outcome of the project and/or an ability to exert a positive or negative influence by the execution or completion of a project or being affected by the work or its deliverables, outputs, or results.

In clinical research, researchers are often faced with questions about the choices that must be made by patients. Research can also be focused on assisting the process of program development. In either instance, the underlying motivation remains the same: to healthcare delivery, to become aware of dysfunctionalities that may exist in healthcare, and to improve the outcomes of proposed changes. It is essential then that research and program processes are assisted by those who are most directly affected by proposals, i.e., the patients themselves. Central to the process of encouraging stakeholder involvement therefore is a basic assumption that patients have the right to make the best decisions about their own health care.

Stakeholder engagement versus stakeholder management (ShM): in recent years, the term “*stakeholder engagement*” (ShE) has become widely used in applied clinical research and new program development. An important reason for this is that it has been repeatedly shown that critical health issues, which are often known to the patients or research subjects themselves, may not have been addressed in the original research or program proposals (8). Stakeholder engagement is a bidirectional process. It begins when the researcher communicates and interacts with stakeholders, and ultimately results in informed decision-making concerning the selection, conduct, as well as dissemination of research findings in order to achieve a desired outcome and enhance accountability (9, 10). Stakeholder engagement is thus differentiated from one-way communication processes that seek to influence groups to agree with a decision that has already been made.

The obligation to serve all stakeholder interests is often called *stakeholder management* (11, 12). The main distinction between *stakeholder management* and *stakeholder engagement* largely rests on the extent to which stakeholders are involved in the decision-making processes. The process of engagement varies across different research programs, but is highly noticeable in complex, multidisciplinary research.

A stakeholder analysis is a process, which provides insights into, and understanding of, the interaction between a project and its stakeholders. In other words, the process of listing, classifying, and assessing the influence of these stakeholders in a project is termed a stakeholder analysis. Stakeholder analysis systematically gathers and analyzes both qualitative and quantitative information thereby to determine whose interest should be taken into account throughout the project. One of the first tasks that a clinical project manager must undertake is to identify how stakeholders can make the greatest impact on the research project or program change, which is being contemplated. The function of stakeholder analysis is to produce an awareness of who will be affected by the project and who can contribute to making the project more successful. The stakeholder analysis, which is usually undertaken at an early stage of planning, is an integral part of risk and reward assessment activities.

It is essential for maximal project effectiveness that managers be committed to the basic philosophy of stakeholder involvement. Project managers must communicate and impart what they see as their goals but also seek to encourage participation by stakeholders so that their perspectives are included in decision making.

The process of identifying, engaging stakeholders must begin well in advance so that dialog is seen to play an important part of project implementation; no decisions should be already made before commencing stakeholder engagement on project-related issues.

## Benefits of Stakeholder Engagement

Well managed projects, although long and complex, create long-term economic gain and social values meaning that proper use of taxpayer’s money. When done correctly, stakeholder engagement provides opportunities to further align clinical research practices with societal needs, values, and expectations, helping to drive long-term sustainability and stakeholder interests.

Stakeholder engagement is intended to help administrators fully realize the benefits of applying community and patient interest in hospital programs, and to ensure that research and program changes benefit those who are most directly affected.

The stakeholder focus group is a communication medium through which the opinions of individuals or groups of individuals who are impacted by the research can be elicited. Focus groups can also serve to clarify each stakeholder’s role and responsibilities, as well as promoting an overall understanding of the project requirements. Such processes also provide stakeholders with an environment in which they can express their opinions and feel that they have been heard.

In a series of related manuals the Patient-Centered Outcomes Research Institute (PCORI) (13) has provided a group of examples of how hospitals and medical clinics can encourage stakeholder involvement, in various research projects or programs whose aim was to improve the quality of medical services.

It can be seen from one of our case studies (see Appendix) that stakeholders can make meaningful contributions to a project when opportunities are structured to encourage their participation. The process of encouraging stakeholder participation is referred to as stakeholder management.

## Requirements for Stakeholder Management

Stakeholder management involves the processes of identifying (both internal as well as external) stakeholders; assessing stakeholders’ skills, knowledge, and expertise; determining stakeholders’ requirements; determining stakeholders’ interests and expectations; determining stakeholders’ communication needs; addressing stakeholders’ issues and concerns as they occur; maintaining a positive relationship and communicating with stakeholders throughout the project; identifying stakeholders’ influence-controlling strategies; making sure that stakeholders are involved in the project at the required level throughout the project; and confirming continuous interactions with the stakeholders. In the area of clinical research patients and other stakeholders such as physicians, clinicians, nurses, and others have critical roles to play. Clinical researchers at the outset of research need to ask for patient participation in the development of research questions. Researchers need to find out the exact characteristics of study participants and to define what the nature of the research outcomes should be. In this process, contributions from patients are helpful and often critically important for project success. The process of carrying out research also involves measuring the results of research interventions and monitoring the progress of the research, especially in terms of whether or not it is being directed toward the initial intentions of the research. Finally, patients, who are often very closely connected with the target populations of the research, have a direct perspective on how the targets of the research will respond to the research recommendations, and therefore, can provide useful inputs for insuring its relevancy.

## Project Stakeholder Management Processes

The PMI identifies four key processes that are associated with the stakeholder management knowledge area in initiating, planning, executing, and monitoring and controlling process groups (7) (Table 1).

**Table 1:** **Four project stakeholder management processes and key outputs**.

Processes	Process groups	Detail	Key outputs
1. Identify stakeholders	Initiating	This is the process of identifying all people or organizations impacted by the project and documenting relevant information regarding their interests, expectations, involvement, and influence on project success	Stakeholder register
2. Plan stakeholder management	Planning	This is the process of defining an approach to managing stakeholders throughout the entire project life cycle as per their interest, importance, impact, and influence over the project	Stakeholder management plan
3. Manage stakeholder engagement	Executing	This is the process of meeting and exceeding the Stakeholder stakeholders’ expectations by continuously communicating with them, clarifying and resolving their issues, addressing their concerns, and improving project performance by implementing their change requests	Issue log Change requests
4. Control stakeholder engagement	Monitoring and controlling	This is the process of evaluating and monitoring overall stakeholder relationships and ensuring stakeholders’ appropriate engagement in the project by adjusting plans and strategies as required	Work performance information Change requests

## Identify Stakeholders

This entails identifying all people or organizations impacted by the project and documenting relevant information regarding their interests, expectations, involvement, and influence on project success. In the hospital setting, the stakeholders are usually the patients, but can also be healthcare professionals and the families of patients. Examples of stakeholders are given in Table 2.

**Table 2:** **The stakeholders can be categorized or classified in many different ways for different purposes**.

Examples of stakeholders in a clinical research setting	
External stakeholders	Internal stakeholders
Board of directors	The project team
Community	
Consultants	Consultants co-principal investigators (co-Pls)
Customers (patients/patient groups)	Clinical research associates (CRAs)
Government agencies	Clinical research coordinators (CRCs)
Healthcare stakeholders	Functional manager (FM)
Industry partners	Medical directors
Legislators or policy makers	Operation manager (OM)
Media	Other clinicians involved in the project
Non-governmental organizations (NGOs)	Portfolio Manager (PfM), Program manager (PM)
Non-profit organizations (NPOs)	Project manager (PM)
Other businesses	Project management office (PMO)
Regulatory agencies	Principal investigators (PIs)
Research partners	Subject matter experts (SMEs)
Scientific communities	Consultants
Sponsors – financial institutions (public and private consultants)	
Subject matter experts (SMEs)	
Vendors or suppliers	

*For example, primary stakeholders (e.g., users of the products or services) or secondary stakeholders (they may not be the end users, however, have some other relationship)*.

Throughout the project the following critical tasks should be carried out.

All internal and external stakeholders should be identified. These will usually be the patients but often will include the patients’ family members, healthcare providers, or program administrators.

Stakeholders’ interests, requirements, and expectations should be identified. Obviously, patients are interested in the effects of proposed program changes or research outcomes on their health and well-being, but may have additional interests such as hoping to improve their employment prospects, or expanding their range of capabilities. Clinical researchers and administrators should be alert to these concerns and take appropriate steps to address them. It has been found, for instance, that stakeholder views at the beginning of a program evaluation process may be provisional or may change as a result of additional information. Additionally, stakeholders’ interests may change over time. In one study, the results of pre-workshop and final workshop voting often differed, suggesting that prioritization efforts relying solely on requests for topics from stakeholder groups without in-person discussion may provide different research priorities (14). Thus, efforts should be made to audit the evolving nature of stakeholders’ expectations and preferences through structured methods.

All stakeholders’ levels of influence should be determined. It is often the case that patients and other beneficiaries of program development have talents and skills that may not be reflected in records of formal education or social standing. Certain personal traits, which patient stakeholders may possess, such as communication skills or life experience, could nevertheless prove invaluable for achieving project goals.

A communication plan for the stakeholders should be determined. Patient stakeholders may not always be familiar with or comfortable in using traditional channels of communication in large organizations. As noted by Lavallee et al. (15), the increasing availability of mobile technology, social media, internet venues, and electronic devices has multiplied the communication options for many, but carries with it the risk of increasing the quantity of participants while reducing the depth of involvement. Often, the use of focus groups or small informal meetings can be used to increase the quality of communication or to elicit participation from those who might otherwise be reticent about expressing their views. Reviews of methods of communication for engaging stakeholders have concluded that a combination of approaches probably yields the best results. Methods such as voting or using ranking procedures such as the analytic hierarchy process (16) and other structured techniques are best for establishing research priorities, whereas in-person methods are best for clarifying ideas and generating ideas (17). Repeated exposure to these experiences be useful for identifying patient stakeholders’ core concerns and for acclimatizing them to organizational communication.

Stakeholders’ expectations and influence over the project should be managed. Reality checks are important for balancing patients’ idealistic expectations and the necessity of dealing with the challenges of getting things done through institutions. Program administrators must identify patient stakeholders’ strengths and channel these for optimal organizational impact.

Depending on their complexity, size, and type, most projects have a diverse number of internal and external stakeholders at different levels of the organization with different authority levels.

Stakeholder identification is a dynamic and sometimes difficult process, and the influence of a stakeholder may not become evident until later stages of a project. And, sometimes projects evolve so that solving unseen problems emerges as a critical task. It is essential to identify as many as stakeholders as possible at the beginning of the project and classify them according to their level of interest, influence, importance, and expectations at the earliest stages of the project as much as possible (Table 3). The identification of the relevant stakeholders is not only a core necessity but also poses a significant challenge. For example, under cost constraints, it might not be possible to identify all external stakeholders (18). On the other hand, stakeholders who are missed out during the identification process might have special requests to be fulfilled. This could potentially delay the project completion or escalate the cost as their requirement needs to be fulfilled. Additionally, as Bryson (19) pointed out that the failure to attend to the information concerns of stakeholders clearly is a kind of flaw in thinking or action that too often and too predictably leads to poor performance outright failure or even disaster.

**Table 3:** **Stakeholder management strategy**.

Stakeholder	Classification	Potential strategies for gaining support or reducing obstacles
Stakeholder 1	Resistor	Notify the sponsor about the potential negative impact of the stakeholder
		Arrange a meeting with this stakeholder and invite the sponsor to discuss project objectives
		Explain the benefits of the project to the stakeholder
		Try to gain commitment from the stakeholder on the resources and deliverables in the presence of the sponsor
Stakeholder 2	Neutral	Ask the stakeholder to join the project management team and be an active member of the project
Stakeholder 3	Not Supportive	Find out from others who have experience with this stakeholder about how to Work with this person
Stakeholder 4	Difficult	Find out from others who have experience with this stakeholder about how to Work with this person
		Identify requirements clearly and get approval
		Send regular updates

As per the PMBOK^®^, the “Identify Stakeholders” process has the following inputs, tools and techniques, and outputs: 
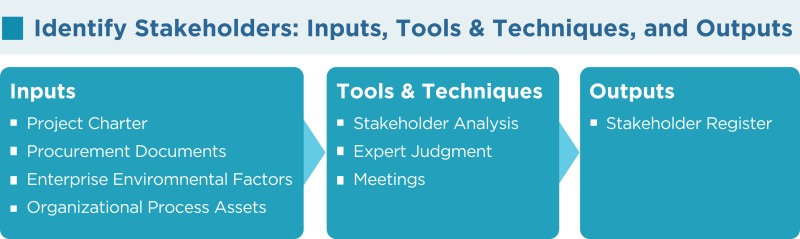
 
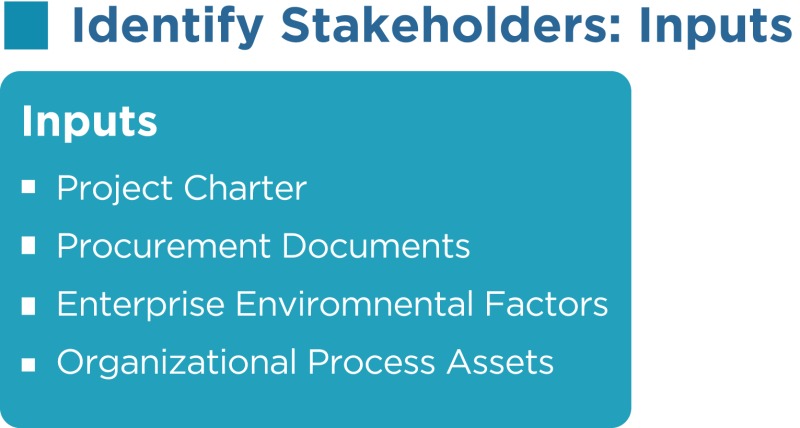


### Project charter

The project charter gives an overall picture of the project as well as describing some of the stakeholders and their interest in the project along with their requirements.

### Procurement documents

If a project is based on an established contract or the result of a procurement activity, the parties in that contract are key project stakeholders. Other relevant parties such as suppliers, legal parties, and people who will execute the contract should also be considered as part of the project stakeholders list.

### Enterprise environmental factors

Hospital culture and structure, and other factors may influence the identify stakeholders process.

### Organizational process assets

To benefit from previous experience those in charge of developing proposals should carefully review the efforts of earlier projects. The stakeholder register template, lessons learned, and the stakeholder registers from previous projects may influence the identify stakeholders process.


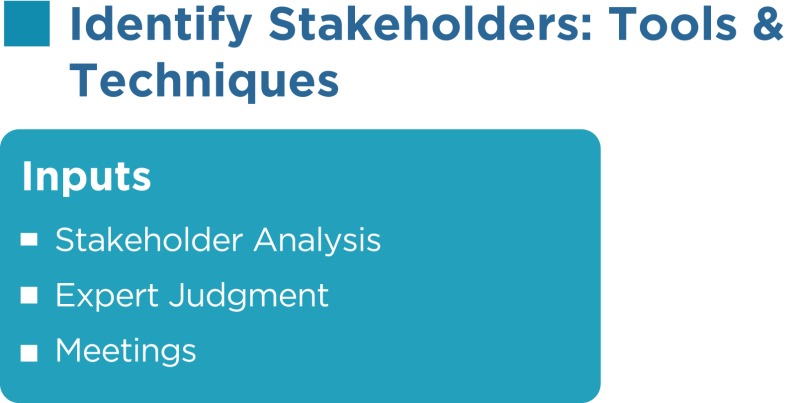


### Stakeholder analysis

It is not possible to treat all stakeholders equally in the project, and they are given different priorities with respect to their interests, expectations, and influence on the project. Stakeholder analysis is a process of systematically gathering and analyzing all relevant quantitative and qualitative information about the stakeholders in order to prioritize them and determine whose interests should be taken into consideration throughout the project.

**Figure 1:**
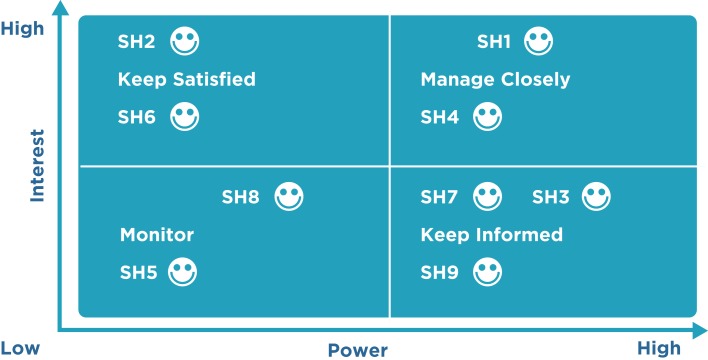
**Stakeholder mapping: the power versus interest grid**. The gird shows stakeholders on a two-by-two matrix showing the strategies to be employed to engage and manage them. Power/interest grid model shows the grouping of the stakeholders based on their level of authority (“power”) and their level or concern (“interest”) regarding the project outcomes. Identifying and classifying the stakeholders is pivotal as it helps to develop appropriate strategies to effectively engage and manage all the stakeholders involved in a particular project. This also provides a clear-cut strategy and action-oriented and workable plan to interact with the all the stakeholders in an effective manner so as to minimize the resistance and maximize the support. A project is as successful as the stakeholders think it is. The details of power versus interest grids are found elsewhere (25).

As per PMI, stakeholder analysis is performed by the following steps:

Step 1: all potential project stakeholders and their relevant information, such as their roles, interests, knowledge levels, expectations, and influence levels should be identified.
Step 2: the potential impact or support each stakeholder can contribute should be identified. As per the PMBOK^®^, there are several classification models below:
  - Power/interest grid: this is based on the level of authority or power and the level of concern or interest that a stakeholder has regarding the project outcome (Figure 1).
  - Power/influence grid: this is based on the level of authority or power and active influence a stakeholder has.
  - Influence/impact grid: this groups stakeholders based on their involvement or influence and their ability to affect changes to planning or execution (impact).
  - Salience model: this addresses a stakeholder’s power or ability to impose their will, urgency, or need for immediate attention from the team and legitimate involvement in a project.
Step 3: in order to influence the stakeholders to enhance their support and to mitigate potential negative impacts, the way in which key stakeholders are likely to react or respond in various situations should be assessed.

Stakeholders who have greater power or influence and a strong interest in the project should be managed closely and continuously updated. Stakeholders who have significant power but low interest in the project should be kept informed about the project. Stakeholders who have low power and low interest should be monitored, and stakeholders who have low power and high interest should be kept satisfied.

### Expert judgment

Judgment and expert opinions can be gathered to identify stakeholders, usually from the senior management. These resources can include project team members, project managers from similar projects, subject matter experts, industry groups and consultants, and other units within the hospital or research setting.

### Meetings

Profile analysis meetings with team members and the sponsor will be beneficial for identifying stakeholders and their knowledge, potential roles, importance, impact, interest, and expectations in the project.


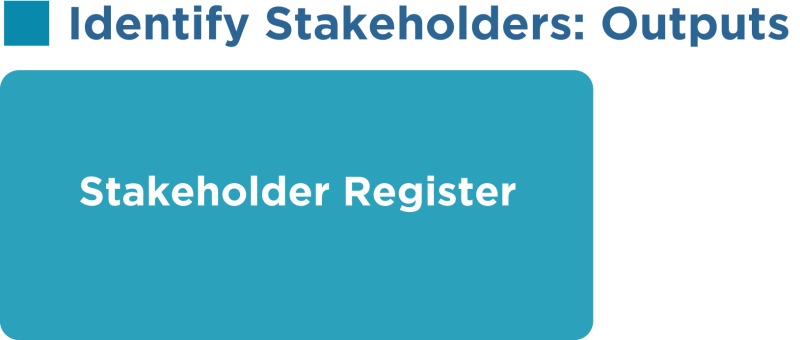


### Stakeholder register

This contains all details related to the identified stakeholders including but not limited to

- Stakeholder classification: stakeholders can be classified in many different ways. For example, primary (users of the products, services, or results) or secondary (may not be the direct users, but have some influential relationship), Internal/external, neutral/resistor/supporter/hard to hear, and so on.
- Identification information: name, title, location, organization, role in the project, position, and contact information.
- Assessment information: key requirements and expectations, potential impact, importance, and influence on the project.

A project manager may publish the stakeholder register with other project documentation or keep it in reserve for personal use only (Table 4).

**Table 4:** **A snapshot of a stakeholder register: stakeholder register is a project document, which is an output of identify stakeholders process**.

Stakeholder register													
Project ID #			Project title #										
ID	Name	Title	Organization	Contact info	Spectrum of stakeholder position (in a scale of 1–5)				Classification	Key expectation	Information needed	Role(s) in project	Action plan (ways to manage expectations – action to maximize support or reaction to minimize disruption)

					Knowledge Level	Interest Level	Influence Level	Power Level

JD101	Dr. John Doe	Co-investigator	MIT	617-222-1111	1	4	5	4	External and Supporter	1. Functional module 2. Reporting tools	1. Status update 2. Issue logs	Verify proposed budget

JD102	Mrs. Jane Doe	Telephone screener – patient recruitment	NYU	347-222-3333	4	5	4	1	Neutral	Monthly recruitment target	Weekly reports	Assist with patient recruitment plan

Evaluators:								Location.
Signature:								Date.
Signature:								Date.					

*Stakeholder Register will be an important input to the plan stakeholder management process, as well as several other planning processes, which includes plan communication management. A stakeholder register may contain key information such as the identification, assessment, and classification of project stakeholders. A snap shot of stakeholder analysis to show how the stakeholders and their interest areas mapped onto a matrix. The stakeholder’s position might vary from a supporter (S); moderate supporter (MS); neutral (N); moderate resistor/opponent (MR or MO); and resistor/opponent (R or O). Identifying and analyzing the stakeholders are crucial for a project success*.

## Plan Stakeholder Management

The plan stakeholder management process defines an approach for managing stakeholders throughout the entire project life cycle as per their interest, impact, importance, and influence over the project. It defines the strategies for building close relationships with stakeholders who can benefit the project and for minimizing the influence of stakeholders who may have a negative impact on the project.


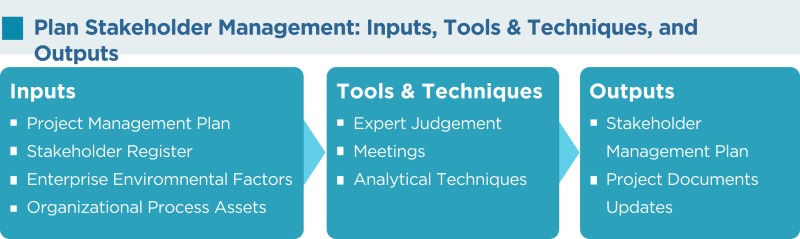


This process is iterative and should be reviewed on a regular basis as the required level of engagement of the stakeholders’ changes in the project.

As per the PMBOK^®^, the Plan Stakeholder Management process has the following inputs, tools and techniques, and outputs: 
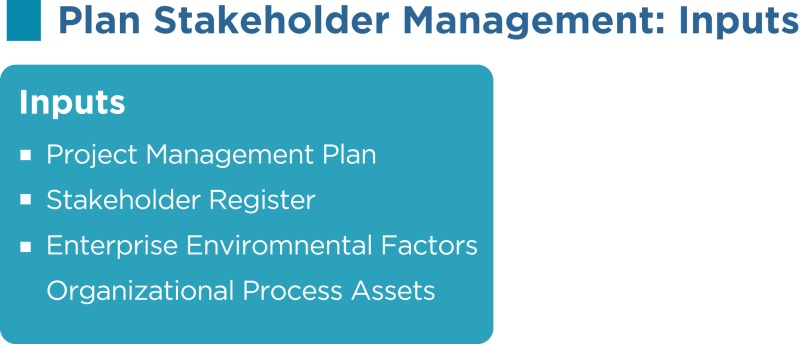


### Project management plan

Components of the project management plan (PMP) such as the human resource management plan, staffing management plan, communications management plan, change management plan, and others are used in developing the stakeholder management plan (SMP).

### Stakeholder register

This contains all details related to the identified stakeholders, including identification information, assessment information, and classification.

### Enterprise environmental factors

All environmental factors within the hospital or clinical research facility, including its culture and history of the organization, are used.

### Organizational process assets

All organizational process assets, especially lessons learned and historical information are used.


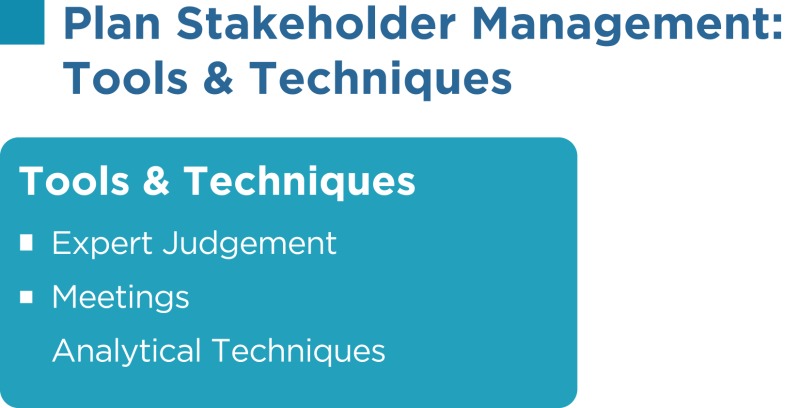


### Expert judgment

Judgment and expert opinions can be gathered from senior management, project team members, identified stakeholders, project managers from similar projects, subject matter experts, industry groups and consultants, other units within the organization, and other people to identify the level of involvement required from each stakeholder at various stages of the project. However, it is possible that expert judgment can be mistaken when possible expert judgment must be balanced with input from the stakeholders themselves.

### Meetings

Meetings with team members and the sponsor will be beneficial for identifying the level of engagement required from each stakeholder.

### Analytical techniques

Various analytical techniques are used for identifying the required level of stakeholder engagement. These techniques take into consideration stakeholder sensitivity to project goals and personal orientations such as being unaware, resistant, neutral, supportive, or providing leadership.

### Stakeholder engagement assessment matrix

The stakeholder engagement assessment matrix (SEAM) is used to assess the current and desired state of engagement of a stakeholder for the current phase of the project (Table 5).

**Table 5:** **Stakeholder engagement assessment matrix (SEAM): please note that the current and desired engagement level of key stakeholders expect to change as the project progresses and develops**.

Project stakeholders	Project stakeholders	Project stakeholders	Project stakeholders	Project stakeholders	Project stakeholders
Stakeholder 1		C			D
Stakeholder 1	C			D	
Stakeholder 1		C		D	
Stakeholder 1				C, D	
Stakeholder 1				C	D
Stakeholder 1			C	D	

*C, current state; D, desired state*.

The SEAM illustrates that only Stakeholder 4 is engaged in the project at the desired state. The project manager should consider additional communication and further actions to bring all other stakeholders to the supportive and leading states.

Stakeholder engagement is critical to project success; thus, required actions and communication should be planned to minimize the gap between the desired level of engagement and the actual level of engagement.


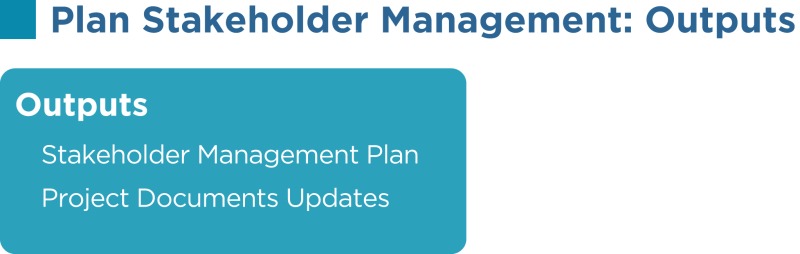


### Stakeholder management plan

Stakeholder management plan, which is a subsidiary plan of the PMP that defines the processes, procedures, tools, and techniques to effectively engage stakeholders in project decisions and execution on the analysis of their needs, interests, and potential impact (7). The SMP can be formal, informal, highly detailed, or broadly framed based on the needs of the project. The SMP typically describes the following:

- information needs of each stakeholder or stakeholder group;
- stakeholder communication requirements;
- format, method, time frame, and frequency for the distribution of required information to the stakeholders;
- person responsible for communicating the information to the stakeholders;
- methods of refining the SMP;
- required engagement level of the stakeholders at various stages of the project;
- stakeholder management strategy that defines an approach to manage stakeholders throughout the entire project life cycle. It defines the strategies to increase the support of the stakeholders who can impact the project positively and minimize the negative impacts or intentions of the stakeholders who can negatively impact the project.

The portion of the plan that contains sensitive information such as stakeholders’ personalities and attitudes, negative impact that stakeholders may cause, or other factors is not usually published and is kept in reserve by the project manager for personal use only.

### Project documents updates

Project documents such as the project schedule, stakeholder register, and others may be updated.

## Manage Stakeholder Engagement

The Manage Stakeholder Engagement process is focused on meeting and exceeding the stakeholders’ expectations by continuously communicating with them, clarifying and resolving their issues, addressing their concerns, and improving the project performance by implementing their change requests.

As per PMI, the project manager is responsible for managing the stakeholders’ expectations. Meeting the stakeholders’ expectations increases the probability of project success by enabling the stakeholders to be active supporters of the project, drastically reducing unresolved stakeholder issues, and limiting disruptions in the project.

As per the PMBOK^®^, the Manage Stakeholder Engagement process has the following inputs, tools and techniques, and outputs: 
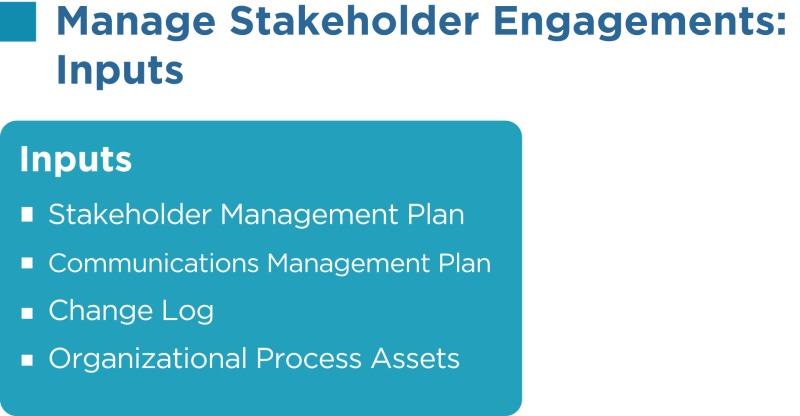


### Stakeholder management plan

Within the research context, the SMP identifies information needs, communication requirements, required engagement level at various stages of the project, stakeholder management strategy, and other factors to identify and manage stakeholders throughout the entire project life cycle.

### Communications management plan

The communications management plan is a subsidiary of the PMP. It can be formal, informal, highly detailed, or broadly framed based on the needs of the project. The communications management plan typically describes the following: purpose for communication; Information needs of each stakeholder or stakeholder group; stakeholder communication requirements; format, method, time frame, and frequency for the distribution of required information; person responsible for communicating the information; methods for updating the communications management plan; persons or groups who will receive the information; glossary of common terms; issues/concerns escalation procedures.


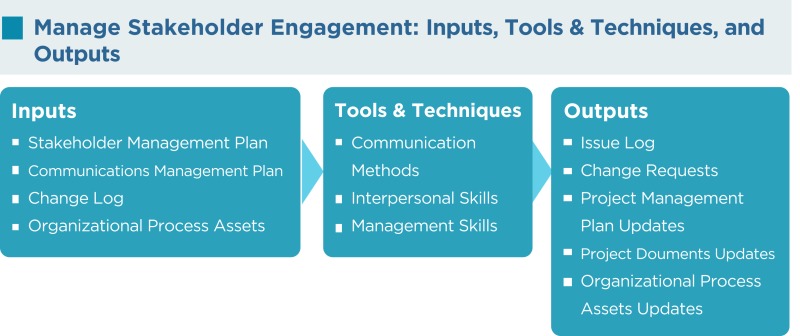


### Change log

A change log is used to document changes that occur during a project. A lot of these changes can impact different stakeholder interests; thus, the change log is reviewed in this process.

### Organizational process assets

Organization communication requirements, issue management procedures, change control procedures, and historical information are used.


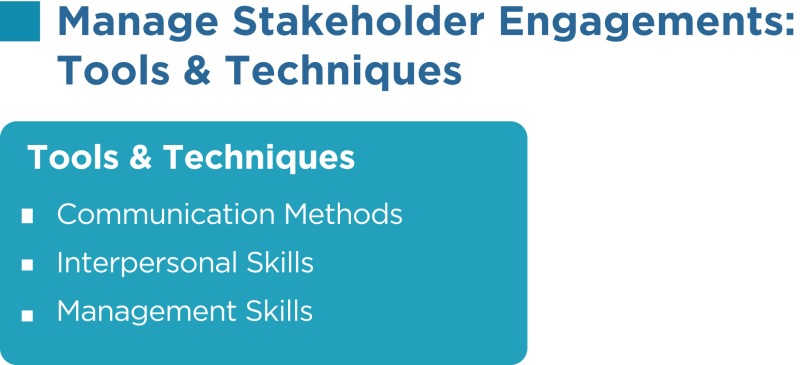


### Communication methods

According to the needs of the project, the methods of communication identified for each stakeholder in the communications management plan are utilized during the manage stakeholder engagement process.

### Interpersonal skills

The project manager applies appropriate interpersonal skills or soft skills to manage stakeholder expectations by building trust and resolving conflict.

### Management skills

Management skills such as presentation skills, negotiation skills, writing skills, and public speaking skills used by the project manager can greatly influence how stakeholders feel about the project.


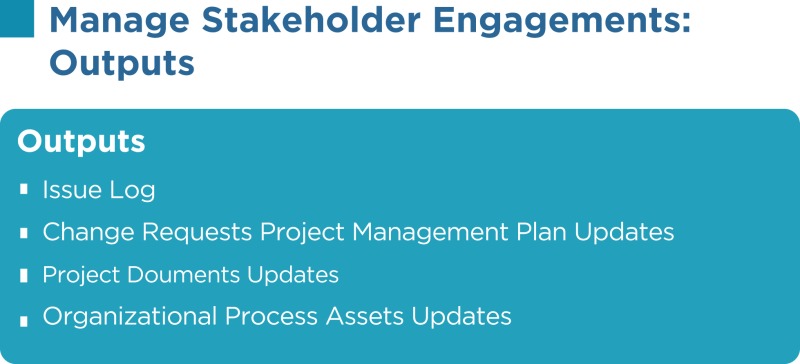


### Issue log

An issue is an obstacle that threatens project progress and can block the team from achieving its goals. An issue log is a written log document to record issues that require a solution. It helps monitor who is responsible for resolving specific issues by a target date. There should be one owner assigned for each issue reported within the project.

### Change requests

Change requests can include a new change to the product or the project, corrective or preventive actions, and other items.

### Project management plan updates

The SMP portion of the PMP is updated as new stakeholders’ requirements are identified, existing requirements are changed, or as a result of addressing concerns and resolving issues of the stakeholders.

### Project documents updates

Project documents that may be updated include, but are not limited to, the following:

- Issue log: this will be updated as resolutions to the current issues are implemented and new issues are identified.
- Stakeholder register: this is updated as stakeholders’ statuses change, new stakeholders are identified, registered stakeholders are no longer involved or impacted by the project, and other factors.

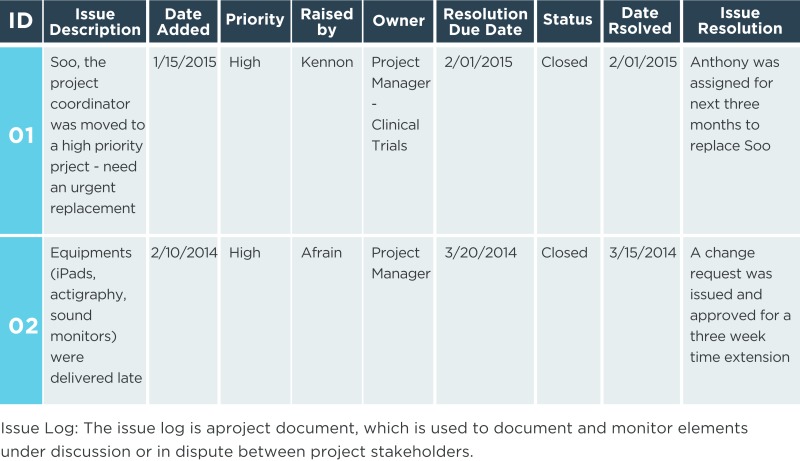



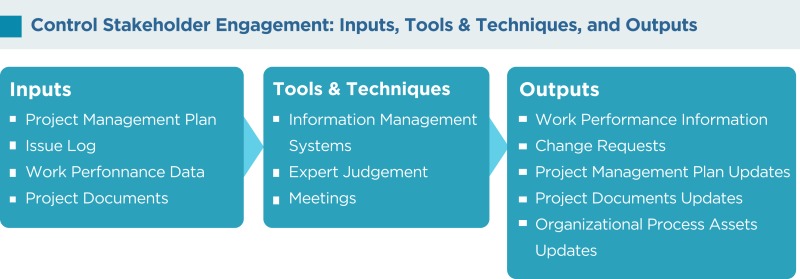


### Organizational process assets updates

Lessons learned from managing stakeholders, feedback from stakeholders, project records, causes of issues, and reasons for corrective actions chosen may be updated.

## Control Stakeholder Engagement

The control stakeholder engagement is the process of evaluating and monitoring overall stakeholder relationships and ensuring stakeholders’ appropriate engagement in the project by adjusting plans and strategies as required. As the project progresses and its environment changes, this process will maintain or increase the efficiency and effectiveness of stakeholder engagement activities.

As per the PMBOK^®^, the Control Stakeholder Engagement process has the following inputs, tools and techniques, and outputs: 
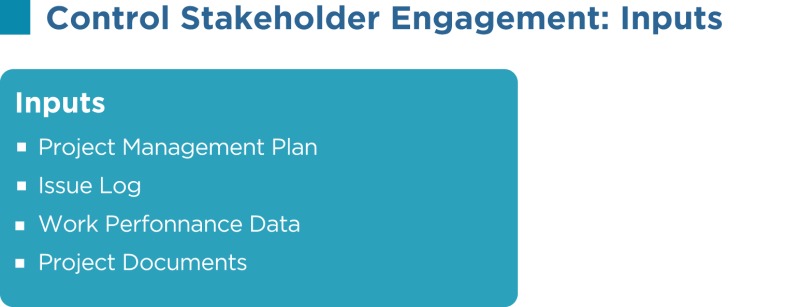


### Project management plan

Components of the PMP such as the human resource management plan, staffing management plan, communications management plan, change management plan, and others are used in controlling stakeholder engagement.

### Issue log

An issue is an obstacle that threatens project progress and can block the team from achieving its goals. An issue log is a written log document to record issues that require a solution. A modified issue log is developed as a result of identifying new issues and resolving current issues.

### Work performance data

Work performance data such as resource utilization, deliverables status, schedule progress, percentage of work completed, number of defects, number of change requests, technical performance measures, costs incurred, quality updates, and other factors are used in this process.

### Project documents

Project documents such as issue logs, the stakeholder register, the project schedule, the change log, and others are used in this process.


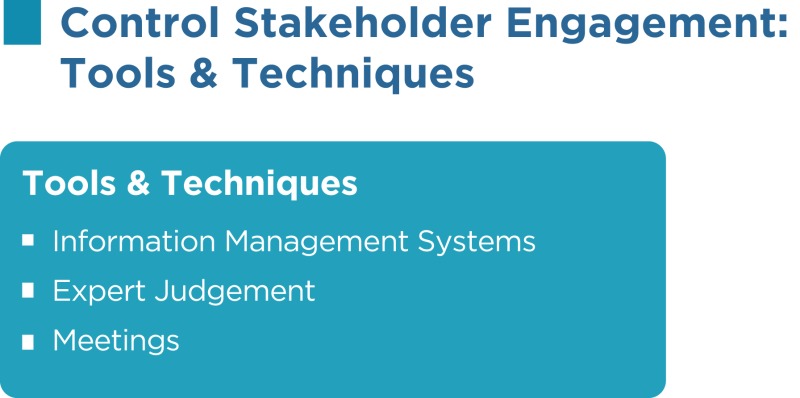


### Information management systems

An information management system is an automated system that can serve as a repository for information, a tool to assist with communication, and a system for tracking documents and deliverables. An information management system also supports the project from beginning to end by collecting and distributing information about cost, schedule, and performance for the stakeholders. Several reporting techniques such as spreadsheet analysis, table reporting, presentations, graphics for visual representations, and others may be consolidated from various systems and communicated to the stakeholders.

### Expert judgment

Judgment and expert opinions can be gathered from senior management, project team members, identified stakeholders, project managers from similar projects, subject matter experts, industry groups and consultants, other units within the organization, and other people to identify new stakeholders, reassess the current stakeholders, and figure out the level of involvement required from each stakeholder at various stages of the project.

### Meetings

Status review meetings with the team, sponsor, and other stakeholders will be beneficial for reviewing information about stakeholder engagement.


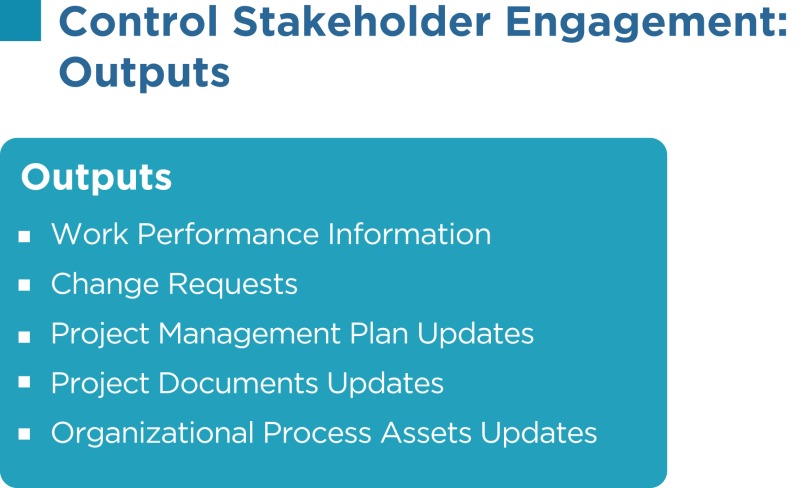


### Work performance information

Work performance information such as deliverables status, change request implementation status, and forecasted estimates to completion are distributed through communication processes.

### Change requests

These are recommended corrective actions for bringing the imminent performance of the project as per the expectations in the PMP and recommended preventive actions for reducing the probability, and impact of future negative project performance will generate a lot of change requests.

### Project management plan updates

Most of the components of the PMP may be updated to reflect changes in the stakeholder management strategy and the approach to effectively control stakeholder engagement in the project.

### Project documents updates

Project documents such as the issue log, the stakeholder register, and others may be updated.

### Organizational process assets updates

Lessons learned from managing stakeholders, feedback from stakeholders, project records, causes of issues, reasons for corrective actions chosen, project reports, stakeholder notifications, and other items may be updated.

## Discussion

While the burden of disease is growing rampantly and disproportionately, the challenge to global health outreach efforts is to prioritize those illnesses, which require immediate attention. The global health equity sorts to prioritize on improving health care and achieving equity in health of people around the world. In this context, researchers from high income countries often study the existing diseases and/or emerging challenges in low income countries in order to gain expertise on the health care needs (20). In this regard, it is essential for overall program effectiveness that representatives of local communities, the stakeholders who will be most impacted by health outreach programs, be invited to provide their insights into which health needs are greatest. The encouragement of ShE and ShM often has a secondary benefit inasmuch as organization’s reputation is subsequently enhanced, which further facilitates organizational effectiveness. The very presence of stakeholders may foster an organizational environment, which encourages relevancy of program objectives to stakeholders’ expectations, a coupling, which in turn contributes to achievement of the project’s goals. Additionally, stakeholders can provide reality checks, which aid in the prioritizing of research objectives, in identifying potentially difficult political issues, and in providing the means to navigate around or to overcome challenges. The experience of stakeholders is thus invaluable for guiding research and achieving program objectives from their early stages in the laboratory to their final clinical application.

Although the process of partnering with stakeholders in clinical research settings is still in its nascent stages, it is anticipated that it will increasingly become accepted and implemented by project managers. In tandem with this process, greater efficiency and transparency will develop in working with stakeholders to meet targets (21). Part of the function of stakeholder analysis is to promote an understanding of stakeholders and to ensure that their expectations are being met. It is anticipated that project heads will increasingly encourage an awareness (ensuring transparency) of who will be affected by the project and who can contribute to making the project more successful.

Stakeholders have unique perspectives and often possess a number of capabilities which they have acquired from life experience. Program developers can derive the maximum benefit from stakeholders if the proper context is established for drawing out this experience. Alternatively, barriers to effective participation by stakeholders can occur if managers remain unaware of stakeholders’ skills, or if they believe that they do not have appropriate knowledge to contribute.

By increasing the acceptability of programs, stakeholders increase the likelihood of their success. Stakeholders play pivotal roles as healthcare advocates or healthcare ambassadors, partners, and/or agents of change. Although stakeholders differ considerably in their expertise and interests, their involvement is pivotal inasmuch as it can facilitate the successful completion of projects. Stakeholder participation can (a) improve relevance; (b) promote visibility and research transparency; (c) accelerate and translate the research findings to real-world challenges; (d) enhance greater project acceptance as confidence derived in the decisions made during the project’s milestone developments. Similarly, the project’s final outcome can only be considered successful when it is acknowledged by its key stakeholders.

Due to the broad range of ways in which stakeholders can influence program development, it is essential that their behavior be closely monitored, and modulated if necessary. One of the advantages of the described system of viewing the management of stakeholder engagement is that it documents many processes that have taken place. Future efforts to manage this type of engagement can therefore benefit from established experience.

In a nutshell, as Wheeler et al. (22) pointed out, “a truly stakeholder-responsive approach demands the acceptance of multiple stakeholders and requires that an organization develop a tolerance for ambiguity together with the sensitivities and capabilities needed to inspire trust with diverse and sometimes completing interests.”

A balanced assessment recognizes that certain caveats apply in the establishment of stakeholder engagement and management in clinical and research settings. These relate to the unique nature, demands, resources, and implementation issues which every organization has and how these demands can interact with the unique skills and abilities which stakeholders bring to it.

**Figure 2:**
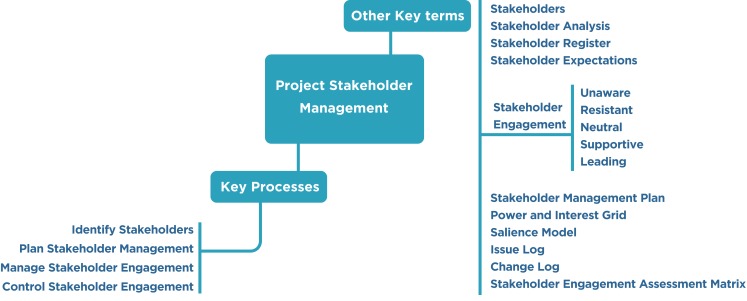
**Project stakeholder management summary**.

Many investigators lack clear or a basic understanding and/or training concerning the stakeholder framework as well as terminologies (Figure 2).

Often a reactive approach is favored over a proactive one for dealing with stakeholder issues is favored over a proactive one. As suggested by Greenwood, the “glorified depiction of stakeholder engagement gives way to the murky reality that engagement of stakeholders can mean many things to many people” (23). In this conceptualization, stakeholders may be viewed merely as actors with whom project managers form arms-length transactions rather making a tightly knitted partnership. This limited perspective may result in a failure to assess, understand the social, spiritual, and environmental needs of stakeholders.

Not every project can require or afford to have a full ShE or ShM system in place as outlined in this review. The main barrier here is that stakeholder involvement, and the fostering of attitudes that encourage this process, require a degree of organizational change (i.e., additional paperwork, more meetings, and more communication), which can be expensive. Hence, the benefits of managing a full program of stakeholder engagement, analysis, and management are only applicable to high impact projects. As Cennamo et al. (24) have noted a stakeholder-committed organization may still act out of self-interest. Thus, while localized programs may recognize and incorporate contributions from stakeholders and the larger community, these activities may continue to serve institutional objectives, which are narrowly focused or even possibly inimical to broader community interests. Additionally, stakeholder preferences are not absolute, but relative, and may also be evolving. Hence, the salience might change frequently across time. Another issue is that there is no generic “one size fits all” strategy for ShM and ShE, rather, the strategy and its execution depend very much on the local stakeholder landscape, as well as the problems that are being addressed. These include the stakeholder assets that are available and the opportunities that exist for their cultivation. Additionally, a barrier to effective participation and the subsequent consensus-building process is that the identified stakeholders may lack appropriate knowledge or skill sets, or believe, correctly or incorrectly, that they do not have appropriate knowledge to contribute and/or the investigators have the knowledge and experience to identify it. Stakeholders might have multiple perspectives and conflicting views, needs, and priorities. These may eventually result in identifying what they perceive to be the “best” or “appropriate” solution in any given situation, although the course of action might completely differ from that of investigators. This, in turn, results in potential “conflicts” and “trade-offs” in terms of project objectives. For example, the conflicting interest among the stakeholders with varied levels of power, importance, interest, and agenda must be managed efficiently. This poses a challenge to novice project managers to experienced investigators. These may potentially limit control mechanisms, and thus impede organizational performance. On the contrary, it is difficult to generate interest and involvement in projects, which are perceived to have little or immediate relevance. Finally and most importantly, while most of the ShE literature emphasizes the positive benefits of stakeholder engagement, it less frequently addresses the potential costs and risks with the adoption of a stakeholder perspective.

Above all, it is impossible to engage with stakeholders and to do stakeholder management in an authentic and effective way without dealing with the multiplicity of ethical issues that arise. These issues arise, first of all, because the stakeholders’ interests can conflict along key ethical dimensions. Therefore, engaging with them must be sensitive to the rights of the parties involved, as well as to the overall harms and benefits, which accrue from managerial action. Second, it is not always apparent when there may be conflicts of interests or hidden advantages or hidden disadvantages among key stakeholders and decision makers. Obviously, these conflicts must be disclosed, and many organizations have specific procedures for handling such conflicts. However, given the nature of the decisions that are to be made, managers must be willing to accept that effective stakeholder management places them squarely in the realm of ethical decision making.

It is not always possible to anticipate all of the ethical issues and conflicts, which may develop in such a multi-stakeholder environment. Therefore, the traditional method of assigning responsibility for solving these problems to an ethics committee does not always work. Clinical project managers must be willing to make choices based on both good ethics (based on PMI’s code of ethics and professional conduct) and on the overall purpose and values of their institution that is best for all stakeholders (27). While some ethical issues can be anticipated at the start of the project, all should be subjected to discussion among the project stakeholders to find the best possible course of action.

Assuming that the challenges reviewed above can be overcome, additional “higher order” issues will emerge. These will consist of how to best promote the operational adaptability, viability, and implementation of the changes in an acceptable timeframe. Project managers will need to ask if the benefits of managing a full stakeholder analysis are really greater than the costs associated with it. Efforts will also need to be directed toward retrospective analysis, i.e., did real cases go badly because the stakeholder views were not sought out? The difficulty of these questions varies in different clinical settings but it is essential that they be resolved for maximum project effectiveness.

In summary, the concept of promoting stakeholder engagement and management is a relatively recent one in the clinical research arena; hence, there are many lessons to be learnt in the coming years. As this is an iterative process, although the current efforts from funding agencies such as PCORI are necessary but are insufficient to respond to the above challenges. All indications are that attempts to meet these challenges will nevertheless provide significant benefits for project management effectiveness.

## Conclusion

From a clinical standpoint, stakeholder engagement and management is pivotal to the development and deployment of community-oriented national and global health initiatives. The ultimate purpose of such engagement is the efficient use of time, money, and resources thereby positively impact existing and/or emerging healthcare challenges.

For the purpose of our review, we have followed the guidelines of PMBOK^®^ (7), which provides a common vocabulary to guide the processes. In doing so, our review outlines a systematic model for planning, managing, and implementing stakeholder engagement based on PMBOK^®^ guidelines. Further, the application of the project management knowledge, skills, tools, and techniques can augment the chances of success, even in complex projects.

This review has drawn on the experience of stakeholder engagement in private organizations and government agencies and has argued that the process is equally viable in hospital program development and in clinical research. It has taken the view that the concept of stakeholder engagement and a proper stakeholder management framework is more than a useful adjunct to pursuing project or program goals and is actually pivotal for enhancing organizational success.

These guidelines have been broken down into a number of component parts. It emphasizes that the stakeholders should first be identified, that their interests and expectations should be understood, and that their level of power and influence should be understood as well. A plan for communicating with stakeholders has been outlined and techniques for encouraging their participation and management have been laid out.

## Author Contributions

All authors intellectually contributed to the design, analysis, and interpretation of the results and to drafting the critical review of manuscript. All authors reviewed and approved the final version of the manuscript.

## Conflict of Interest Statement

The authors declare that the research was conducted in the absence of any commercial or financial relationships that could be construed as a potential conflict of interest.

## Appendix: Examples of Stakeholder Engagement

### Statewide Telepsychiatry Initiative

In one of our landmark study by the author (MN), the South Carolina Department of Mental Health partnered with the University of South Carolina School of Medicine, Department of Health and Human Services and 18 predominantly rural hospitals from the South Carolina Hospital Association to establish the statewide telepsychiatry initiative. In this public-private-academic partnership, the psychiatrists were available via teleconference 16 h a day, 7 days a week, to assess and treat patients with mental health issues at hospital emergency departments.

The clinicians called the psychiatrist on duty whenever they have a patient who needed a mental health assessment and/or counseling and provide relevant medical records and details. Through a secure video link, the psychiatrists were able to assess the patient and makes recommendations about needed treatment and follow-up, including referrals to community-based resources.

The advantage of such program was that, it drastically reduced emergency department wait times, inpatient admissions, as well as costs; increased attendance at follow-up outpatient appointments; and generated higher level of satisfaction among patients and clinicians. Additional evidence includes post-implementation surveys that gauge the satisfaction of patients and clinicians with the program.

In such an innovative approach, the stakeholder (State Department of Mental Health, University of South Carolina, Department of Neuropsychiatry and Behavioral Science, Hospital, Physicians and Nurses from the South Carolina Hospital Association, and Patients) engagement proved to be feasible, necessary, and beneficial in clinical environment.

#### Outcome of the initiatives – did it work?

The program has reduced emergency department (ED) wait times, inpatient admissions, and costs; increased attendance at follow-up outpatient appointments; and generated high levels of satisfaction among patients and clinicians.

Shorter ED Wait Times: From March 2009 through 2014, the average waiting time for patients with mental health problems at the 18 participating EDs fell by roughly 50%. Fewer hospitalizations: during the same timeframe, 11% of ED patients assessed by a psychiatrist were hospitalized, half of the 22% admission rate among similarly cared-for patients in South Carolina EDs not offering this program.

Health care utilization: Telepsychiatry consultations resulted in an estimated $1,400 less per mental health patient compared to patients seen in ED’s that did not have telepsychiatry due primarily to the reductions in hospital admissions.

Greater attendance at follow-up appointments: About 46% of patients served by the program attended an outpatient follow-up appointment within 30ădays of the initial ED visit, well above the 16% attendance rate among similar ED patients cared for in South Carolina hospitals not offering the program. Similarly, 54% of patients served by the program attended a follow-up appointment within 90ădays versus 20% among ED patients in hospitals not offering the program.

High satisfaction among patients and staff: More than 80% of patients served by the program reported being satisfied with the process and services received. In addition, 84% of ED physicians and staff believe that the program has improved patient care; 91% report being satisfied with program-related procedures; and 84% express satisfaction with the technology used.

#### How we did it: Planning and development process key steps included the following:

**Decision to focus on telepsychiatry**: The increasing popularity of telemedicine and its potential to bring services to underserved geographic areas made telepsychiatry a logical initial target for these discussions. After reviewing the use of telemedicine in South Carolina and other States, the partners collectively decided to create and implement a telepsychiatry program in hospital EDs.

**Securing of funding for demonstration project**: In 2007, program leaders contacted the Duke Endowment (a non-profit foundation that supports innovative health care programs) for funding a demonstration project. In 2008, the Duke Endowment agreed to provide a $3.7 million, 3-year grant for this purpose.

**Project planning**: Four hospitals agreed to participate in the demonstration project, which launched in March 2009. In advance of this date, the partners and participating hospitals worked together to hire and train six psychiatrists, install and test the video equipment in EDs, and set up an EMR system linking the psychiatrists with the EDs.

**Program expansion**: After the demonstration project went smoothly, program leaders decided in June 2009 to expand the program to three additional sites and have continued to add more sites gradually since that time. From March 2009 through August 2014, more than 20,000 telepsychiatry ED consults have taken place. As of August 2014, 20 hospitals participate, with plans to add 6 more in 2014.

**Standardization of training**: As the initiative expanded, program leaders standardized the training process for psychiatrists and ED staff, as outlined below:

**Psychiatrist training**: Participating psychiatrists complete 6ăh of clinical training by viewing videos prepared by University of South Carolina School of Medicine faculty. Supplemented with handouts, the videos cover child and adolescent psychiatry, adult psychiatry, geriatric psychiatry, addiction psychiatry, risk assessment, and legal issues. Newly hired psychiatrists also undergo peer review every 2ăweeks during their first 3ămonths of employment. In addition, during this initial 3-month period, the supervising psychiatrist meets with other hospital physicians to review and discuss consultations performed by newly hired psychiatrists.

**ED staff training**: ED staffs in participating hospitals watch a video that explains the videoconferencing system and reviews the goals of the program and the training and credentials of the participating psychiatrists. A member of the project leadership team visits each participating ED to discuss questions or concerns that staff might have about the program.

**Ongoing meetings to resolve problems, plan expansion**: Representatives of the partnering organizations and the participating hospitals meet on a quarterly basis to share program-related experiences, resolve any issues or problems that arise, and discuss and plan for expansion to other EDs. Typically, hospital administrators, providers, researchers, and information technology staff come to these meetings; representatives of non-participating hospitals are also welcome to attend to learn more about the program.

#### Resources used and skills needed

**Staffing**: Six full-time psychiatrists and one part-time psychiatrist staff the program, under the supervision of a lead psychiatrist. The program includes several stakeholders namely a director, coordinator, fiscal technician, programmer, and two information resource consultants (subject matter experts; SMEs). Faculty and staff members from the University of South Carolina and Emory University, staff members from the Department of Mental Health and from the South Carolina Office of Research and Statistics also provide support to the program.

**Costs**: Hard data on the program’s total annual cost are not available. Major program expenses consist of staff salaries and the upfront and ongoing expenses associated with the videoconferencing and EMR technologies.

## References

[1] Sox HC Greenfield S Comparative effectiveness research: a report from the institute of medicine. Ann Intern Med (2009) 151:203–5 10.7326/0003-4819-151-3-200908040-00125 19567618

[2] Kellogg Commission. Returning to Our Roots: The Engaged Institution. Kellogg Commission on the Future of State and Land-Grant Universities (1999). p. 1–57 Available from: http://www.purdue.edu/vet/engagement/files/documents/kellogg.pdf

[3] Center for Disease Control and Prevention. Principles of Community Engagement. 2nd ed (2011). 193 p. Available from: http://www.atsdr.cdc.gov/communityengagement/pdf/PCE_Report_508_FINAL.pdf

[4] Conway PH Clancy C Comparative-effectiveness research – implications of the federal coordinating council’s report. N Engl J Med (2009) 2009(361):328–30 10.1056/NEJMp0905631 19567829

[5] Tunis SR Benner J McClellan M Comparative effectiveness research: policy context, methods development and research infrastructure. Stat Med (2010) 29:1963–76 10.1002/sim.3818 20564311

[6] Edward Freeman R Strategic Management: A Stakeholder Approach. Pitman Series in Business and Public Policy. 1st ed Boston: Harper Collins College Div (1984). 275 p.

[7] Project Management Institute. A Guide to the Project Management Body of Knowledge (PMBOK^®^ Guide). 5th ed Newtown Square, PA: USA: Project Management Institute (2013). 589 p.

[8] Negev M Davidovitch N Garb Y Tal A Stakeholder participation in health impact assessment: a multicultural approach. Environ Impact Assess Rev (2013) 43:112–20 10.1016/j.eiar.2013.06.002

[9] Deverka PA Lavallee DC Desai PJ Esmail LC Ramsey SD Veenstra DL Stakeholder participation in comparative effectiveness research: defining a framework for effective engagement. J Comp Eff Res (2012) 1:181–94. 10.2217/cer.12.7 22707880 PMC3371639

[10] Starks H Shaw JL Hiratsuka V Dillard DA Robinson R. Engaging stakeholders to develop a depression management decision support tool in a tribal health system. Qual Life Res (2015) 24(5):1097–105. 10.1007/s11136-014-0810-9 25246185

[11] Bowie NE Werhane PH Management Ethics. Malden, MA: Wiley-Blackwell (2004). 168 p.

[12] Post JE Lee E Preston LE Sachs S Redefining the Corporation: Stakeholder Management and Organizational Wealth. 1 ed Palo Alto, CA: Stanford Business Books (2002). 376 p.

[13] Patient-Centered Outcomes Research Institute. PCORI Funded Projects: Sample Engagement Plans from Methods Portfolio (2014). Available from: http://www.pcori.org/sites/default/files/PCORI-Sample-Methods-Engagement-Plans.pdf

[14] Krishnan JA Lindenauer PK Au DH Carson SS Lee TA McBurnie MA Stakeholder priorities for comparative effectiveness research in chronic obstructive pulmonary disease, a workshop report. Am J Respir Crit Care Med (2013) 187:320–6. 10.1164/rccm.201206-0994WS 23155144 PMC3603554

[15] Lavallee DC Wicks P Cristancho RA Mullins CD. Stakeholder engagement in patient-centered outcomes research: high-touch or high-tech? Expert Rev Pharmacoecon Outcomes Res (2014) 14:335–44. 10.1586/14737167.2014.901890 24661181

[16] Vaidya OS Kumar S Analytic hierarchy process: an overview of applications. Eur J Oper Res (2006) 169:1–29 10.1016/j.ejor.2004.04.028

[17] Guise J-M O’Haire C McPheeters M Most C LaBrant L Lee K A practice-based tool for engaging stakeholders in future research: a synthesis of current practices. J Clin Epidemiol (2013) 66:666–74. 10.1016/j.jclinepi.2012.12.010 23497857

[18] Lindgreen A Kotler P Vanhamme J Maon F A Stakeholder Approach to Corporate Social Responsibility: Pressures, Conflicts, and Reconciliation. Vermont: Gower Publishing Company (2012). 418 p.

[19] Bryson JM What to do when stakeholders matter: stakeholder identification and analysis technique. Public Manag Rev (2004) 6:21–53 10.1080/14719030410001675722

[20] Chu KM Jayaraman S Kyamanywa P Ntakiyiruta G Building research capacity in Africa: equity and global health collaborations. PLoS Med (2014). 11(3):e1001612 10.1371/journal.pmed.1001612 24618823 PMC3949667

[21] Concannon TW Fuster M Saunders T Patel K Wong JB Leslie LK A systematic review of stakeholder engagement in comparative effectiveness and patient-centered outcomes research. J Gen Intern Med (2014) 29:1692–701. 10.1007/s11606-014-2878-x 24893581 PMC4242886

[22] Wheeler D Fabig H Boele R Paradoxes and dilemmas for stakeholder responsive fir in the extractive sector: lessons from the case of Shell and the Ogoni. J Bus Ethics (2002) 39:297–318 10.1023/A:1016542207069

[23] Greenwood M Stakeholder engagement: beyond the myth of corporate responsibility. J Bus Ethics (2007) 74:315–27 10.1007/s10551-007-9509-y

[24] Cennamo C Berrone P Gomez-Mejia LR Does stakeholder management have a dark side? J Bus Ethics (2009) 89:491–507 10.1007/s10551-008-0012-x

[25] Eden C Ackermann F Making Strategy: The Journey of Strategic Management. London: Sage Publications Ltd (1998). 528 p.

[26] Fromson D A Conversation with Cameron Sinclair, CEO of Architecture for Humanity. Available from: http://www.theatlantic.com/national/archive/2011/03/a-conversation-with-cameron-sinclair-ceo-of-architecture-for-humanity/72782/

[27] Project Management Institute. PMI’s Code of Ethics and Professional Conduct. Available from: http://www.pmi.org/en/About-Us/Ethics/~/media/PDF/Ethics/ap_pmicodeofethics.ashx

